# Holistic Medicine III: The Holistic Process Theory of Healing

**DOI:** 10.1100/tsw.2003.100

**Published:** 2003-11-25

**Authors:** Søren Ventegodt, Niels Jørgen Andersen, Joav Merrick

**Affiliations:** ^1^ The Quality of Life Research Center, Teglgårdstræde 4-8, DK-1452 Copenhagen K, Denmark; ^2^ Norwegian School of Management, Sandvika, Norway; ^3^ National Institute of Child Health and Human Development, Office of the Medical Director, Division for Mental Retardation, Ministry of Social Affairs, Jerusalem and Zusman Child Development Center, Division of Community Health, Ben Gurion University, Beer-Sheva, Israel

**Keywords:** quality of life, QOL, philosophy, human development, holistic medicine, public health, Denmark

## Abstract

It is possible to understand the process of healing from a holistic perspective. According to the life mission theory, we can stretch our existence and lower our quality of life when we are in crises, to survive and adapt, and we can relax to increase our quality of life when we later have resources for healing. The holistic process theory explains how this healing comes about: Healing happens in a state of consciousness exactly opposite to the state of crises. The patient enters the “holistic state of healing” when the (1) patient and (2) the physician have a perspective in accordance with life, (3) a safe environment, (4) personal resources, (5) the patient has the will to live, (6) the patient and (7) the physician have the intention of healing, (8) the trust of the patient in the physician, and (9) sufficient holding. The holding must be fivefold, giving the patient (1) acknowledgment, (2) awareness, (3) respect, (4) care, and (5) acceptance. The holistic process has three obligatory steps: (1) to feel, (2) to understand, and (3) to let go of negative decisions. This paper presents a theory for the holistic process of healing, and lists the necessities for holistic therapy restoring the quality of life, health, and ability to function of the patient.

## INTRODUCTION

The process of healing seemingly takes place on two different levels in the organism. Though not completely understood, medical science has a good understanding of the local process of healing that takes place when a specific tissue or organ gets a wound. Healing can also take place on the level of the whole organism, and this is far more mysterious. Biomedical science has been successful in explaining processes on the level of the molecule and the cell, but often unsuccessful in explaining the processes at the level of the organism. It has accordingly not yet been able to explain what happens when patients spontaneously recover or heal completely even from a severe mental or somatic illness, like cancer or schizophrenia. To explain what happens on the level of the whole organism is the objective of holistic medicine[1].

The holistic process of healing seemed to be a complete mystery for medical science, but in the second half of the 20th century, several scientists succeeded in explaining important aspects of this complicated phenomenon. One of the most brilliant was Aaron Antonovsky (1923–94) from the Ben Gurion University in Beer-Sheva, Israel with his model for holistic healing using the famous concept of "salutogenesis"[2,3]. Antonovsky's idea was to help the patient to create a "sense of coherence", an experience in the depth of life, strongly related to the concepts of meaning, understanding, and action. In recovering the sense of coherence, the patient accesses his or her hidden resources and improves quality of life, health, and ability to function at the same time.

Pioneers in the field of holistic medicine have developed different holistic approaches, some fairly successful. Experiments done primarily in the U.S. through the last decades call on a revisited and more concise explanation[4,5,6], a contribution to which we hope to give in this paper.

The explanation presented below accentuates the subjective and global level of the human being. It is holistic and will therefore not deal either with the biology of single cells or with molecules. We are working on explaining the biological mechanisms behind the holistic process of healing. We hope that the scientific community will accept the model in spite of its abstract character, where we take our journey through the life mission theory[7] and not in molecular biology. However strange, the model presented in this paper seems to be of great utility in the daily clinical practice of the family physician, where patients with chronic diseases often need the holistic approach if they are to become better. This model is to be understood as a practical help or tool for the physician, not the final explanation, as we also need the mechanistic explanation at the level of the cells, but that is not given here.

## THE THREE STAGES OF HOLISTIC HEALING: TO FEEL, TO UNDERSTAND, AND TO LET GO

Working directly with the consciousness of the patient is possible because the level of meaning and purpose can be acknowledged by both the patient and the physician in order to work with it and develop[7,8,9,10]. This is often called personal development and is now an increasingly popular trend in our western society. Personal development and holistic healing is also the aim of much alternative, complementary, and holistic therapy.

The human existence can be interpreted as extending from the most abstract level of existence (the consciousness, the spirit, and the soul) to the most concrete level of cells, molecules, and atoms — the physical matter. Taking this as our frame of reference, we can place the phenomena body, feelings, and mind in between the abstract and the concrete level, as shown in Fig. 1[8]. The cells can be found between the level of matter and the level of the body. When these cells are disturbed because of "blockages", illness and suffering arises.

When man experiences unbearable emotional difficulties, these can be solved by repressing emotional pain from the surface of consciousness. This happens by making a negative decision that denies the original constructive intention, which causes the suffering[8]. The existential pain is, together with the whole perception, turned into what is known as a gestalt (a "frozen now"), which is from that moment found as a chronic tension in some part of the mind or the body.

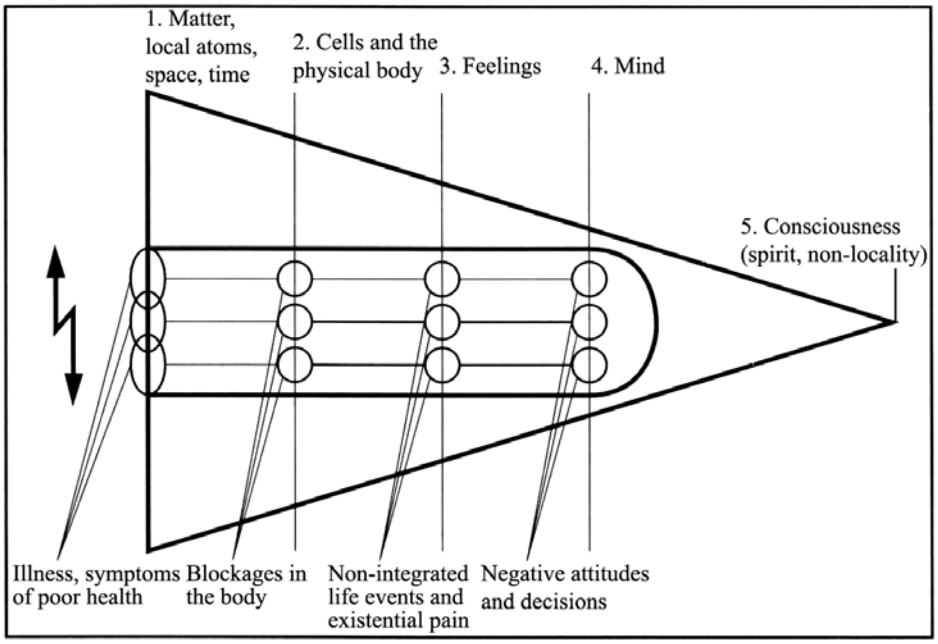

**FIGURE 1.** The holistic process theory of healing can be divided in three steps: "to feel, to understand, and to let go". "To feel" is to set feelings on the body, "to understand" is to set words on the feelings, and "to let go" is to set consciousness on the words. During these three steps, the illness and suffering is treated causally, as the etiology seen from the holistic perspective is the "blockages" in mind and body. The blockages are caused by feelings, suppressed by negative decisions into the tissues of the body. In the optimal process of holistic healing, the three above-mentioned steps occur at the same time.

Working with our patients in holistic therapy, we typically discover these tensions as chronic tightening in the skeletal muscles, but the smooth musculature (such as muscles in the intestines or the uterus) can also hold tensions. Principally, any tissue can hold any kind of tension. The symptoms of these tensions are known as health problems like chronic back pain, chronic stomach pain, and bleeding disturbances without any identified physical cause, or psychosomatic origin. According to this theory, sickness often occurs because emotional pain is suppressed and placed in different tissues in the body, which thereby hold the painful occurrence, the gestalt, until this is processed and reintegrated.

The holistic process of healing is exactly the opposite process of a crisis, creating the problem in the first place; we can identify the same three stages as are found in a crisis, just in the reverse order:

1. The patient initially opens himself up for repressed feelings, feeling them again. Getting rid of the emotional pain is the last step in the crisis.
2. The patient deals with the occurrence in his consciousness and understands his own responsibility about what has happened. Denying the responsibility and escaping the conscious scenario and the painful perception is the intermediate phase of a crisis.
3. The patient perceives the decision that once was made, and understands the inappropriateness of maintaining it. This causes him to let go of it, and heal. Taking this negative perspective or decision is what initially brought the patient into the crisis; of course, this is usually provoked by some unfortunate condition of life.

This three-step model was developed after years of studying the best and most successful kinds of alternative treatment. These were intervening on the levels: body (bodyworks like Rosen therapy), feelings (gestalt psychotherapy), and mind and soul (philosophy of life). The intention was always to help the patient be himself, understand, and take responsibility for his own life. Step 1 was facilitated by body massage and other kinds of physical contact and care, step 2 by psychotherapy and conversations, and step 3 by life philosophical training and reading of insightful books.

For a long time, the three-step model seemed sufficient as these steps really seemed to be what was needed for holistic healing. For several years, we combined bodywork and psychotherapy with philosophical training at the Research Clinic for Holistic Medicine in Copenhagen. The approach worked well for some patients, but most patients were unable to achieve complete recovery and reach the level of full self-expression that was the ultimate goal in the holistic treatment.

Realizing that the alternative therapy gave the patient an experience of getting help without a full recovery made us re-evaluate our approach. We learned that the recovery was sometimes only temporary and that observation forced us to develop the model further, into the holistic theory of healing, presented below. For example: Could a patient with low back pain, treated by holistic bodywork, after a period of feeling better for one month, come back and need a new treatment, and so forth, year after year? Just moving the problem out of the body and into the domain of the patient's feelings did not help the patient, because it was not sufficiently integrated emotionally. Another example was the common experience among psychotherapists, that in spite of rapid and visible progress in the beginning of the therapy, incest victims very seldom got back their normal ability to feel; in spite of many years of therapy their feelings did not heal. Only by making sure that the patient gets through all three stages — in the same therapeutic session or series of sessions — the problem is conclusively solved. When the patient has let go of his negative decisions by the end of therapy, the trauma is completely healed and the experience is like the traumatic event never took place.

To make sure the patient goes through all three steps and obtain real progress in the holistic therapy, it is of advantage that the therapist master all three dimensions of the therapy. The therapist must be holistic in the broadest sense of the word. We learned that if the holistic therapist also is able to give acknowledgment of the soul and spiritual dimension of the patient, and acceptance of the body and sexuality of the patient, he can take the patient into a state of being that we now call "being in the holistic process of healing" or "being in process" for short. When a holistic therapist is able to take his patient into this process, even the most severe traumas seem to go all the way to complete healing.

The entire and complete healing, where the problems are solved by the root of existence, is consequently the goal of holistic medicine. The model has been tested in a sequence of pilot studies[9,10] since 1998, as well as in clinical practice, and it is still being developed. From the clinic follows the example below.

## CASE STORY

As an example of such a patient from our own clinical practice, we can refer to a female patient in her twenties called Anna[11]. Three different men, including her father, raped Anna around 100 times as a child with some of the abuse extremely violent. On arriving at the clinic, she appeared very confused, psychological disturbed, weeping labile, and with poor social functioning. She believed that she was on her way to a nervous breakdown, or maybe even a psychosis at the beginning of the therapy.

In the holistic therapy, she found approximately 200 negative decisions that she successfully let go of. She went through the process, which took two years and approximately 100 therapy sessions of one or two hours, besides thousands of hours of homework. Afterwards, she returned to a normal and healthy emotional state, and could begin to have a natural relationship with men and sex. The therapy occasionally required a substantial holding from several individuals, and during the process she continuously and spontaneously returned to her childhood, until there was no more traumatic material.

During some of the most intense trauma sessions, the patient was in a state of such profound regression that her condition could be described as psychotic. She passed these episodes unproblematically and without any kind of medication, and was capable of taking care of herself between the sessions. After two years of therapy, she entered a calm and stable phase, and was able to make an appraisal of her situation. She gained confidence and self-esteem, and felt that she was in full control of her life. She realized that her intelligence had increased to such a degree that she successfully could study at the university. She started a new life of higher quality, taking into use her intellectual, social, sexual, and many other talents.

## THE HOLISTIC STATE OF HEALING: BEING IN PROCESS

“To be in holistic process” is our designation for the state of holistic healing, achieved by a patient, who is able to trust and receive the holding and processing offered by competent therapists. The patient needs to have the necessary personal resources, in a setting where the intention from both the patient and the physician is the healing of the patient. The process is a “high-energy state of consciousness” often with high arousal, since it has the same intensity as the trauma that originally caused the patient to escape from an overwhelming emotional pain. The result of this holistic process of healing is a spontaneous transfer back to one’s self from the position of the ego[12].

It can be extremely painful and almost unbearable to be in a holistic process, because existential life pains are coming back just as if they had never been deserted. An especially interesting recent finding from our laboratory is that the process does not have to be painful if the patient is supplied with all the necessary resources in the therapeutic session. If the patient is now receiving what historically was missing, the gestalt is not painful, but joyful to confront, as the pleasure of receiving in the present now is greater than the historic pain. We believe this to be an important discovery, since it means that even the most painful traumas can be integrated in a graceful and noble manner into the holistic therapy.

We have identified nine factors that facilitate the process of the patient entering into the holistic process of healing, and staying in it until the process of healing is completed:

1. The physician has a perspective in accordance with life. This comes from a personal philosophy of life that holds life, existence, and every individual soul as sacred and of immense value.
2. The patient has a perspective in accordance with life. The patient appreciates fully the value of his own life, even if this value is not experienced in present time.
3. A safe environment, peace, calmness, and time.
4. Personal resources, rest, tasty food, no crises with family or friends.
5. The patient has a will to live and to be happy.
6. The physician has the intention that the patient will heal.
7. The patient has the intention of healing himself.
8. The patient has enough trust to receive the holding and processing.
9. Substantial and competent holding from the physician, nurse, or other employees.

The five fundamental qualities of holding the patient to “go into process” are:

1. Awareness
2. Respect
3. Care
4. Acknowledgment
5. Acceptance

These qualities correspond with the three existential dimensions of man[11]:

1. Purpose or love – axis: as whole persons we want to give to others. Here the essential is the relationship to other people and what we have to offer (our purpose in life)[11]. The holding need is acknowledgment of our soul and talents.
2. Power – axis: body, feelings, and mind. Here the essential is our consciousness and survival. The holding needs are awareness, respect, and care.

3. Gender and sexuality – axis. Here the essential is pleasure and the ability to enjoy. The holding need is acceptance of body and sexuality.

## THE HOLISTIC PROCESS OF HEALING THE EXISTENCE

If our life is viewed from the perspective of the life mission or purpose in life, the process of holistic healing can be understood in a very simple way. In our natural condition, we live in a balance between "to be" giving us happiness and "to do" giving us often severe emotional and existential pain. Being is in essence a wonderful thing, happiness is an intrinsic factor in life; doing is mostly connected with trouble, effort, failure, and learning. In our natural state of being, life is a dynamic condition in which our existence can be presented as an energy-filled and dancing spring (Fig. 2), the energy of our life — of our being — coiled around our purpose of life, the source of our doing.

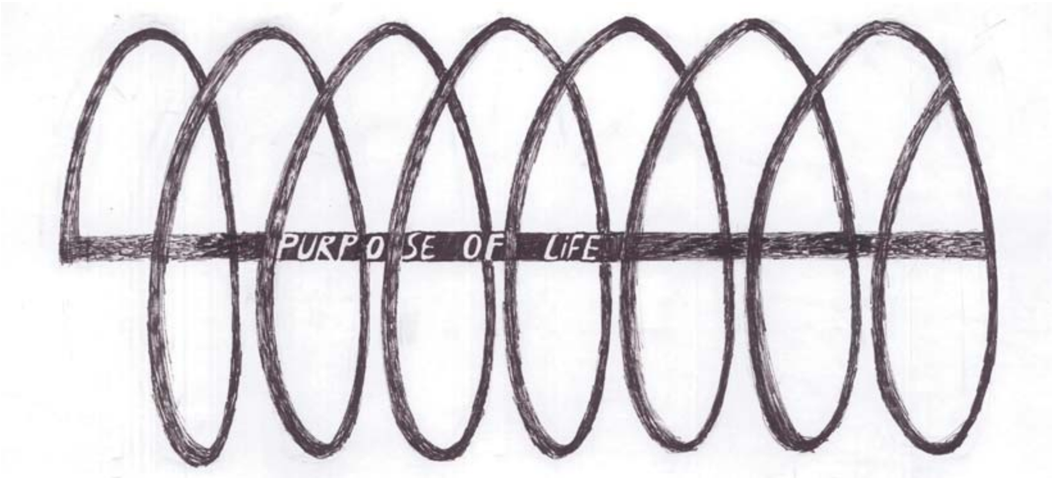

**FIGURE 2.** In our natural condition our existence can be compared with an energy-filled and dancing spring.

When our existential needs are not fulfilled, and especially when we feel that our survival is threatened, which gives us the highest intensity of pain, we may make one or several decisions that modifies our existence. This is done to get what we want, and to survive. Our decisions now stretch the spring, and bind the energy that previously was dancing freely around. As we make more and more negative, existential decisions through life, we move further and further away from our natural state of being (Fig. 3). Psychologically we are loosing the contact with our genuine selves. Mentally we might loose the ability to observe reality from different perspectives, or maybe even our psychological health. Emotionally we might loose the ability to feel. Bodily we might loose our physical health. Sexually we might loose our ability to engage, feel passion, and take pleasure. Spiritually we loose our sense of coherence and meaning. The holistic process of healing our existence brings all of this back, together with our quality of life, health, and functional ability in general.

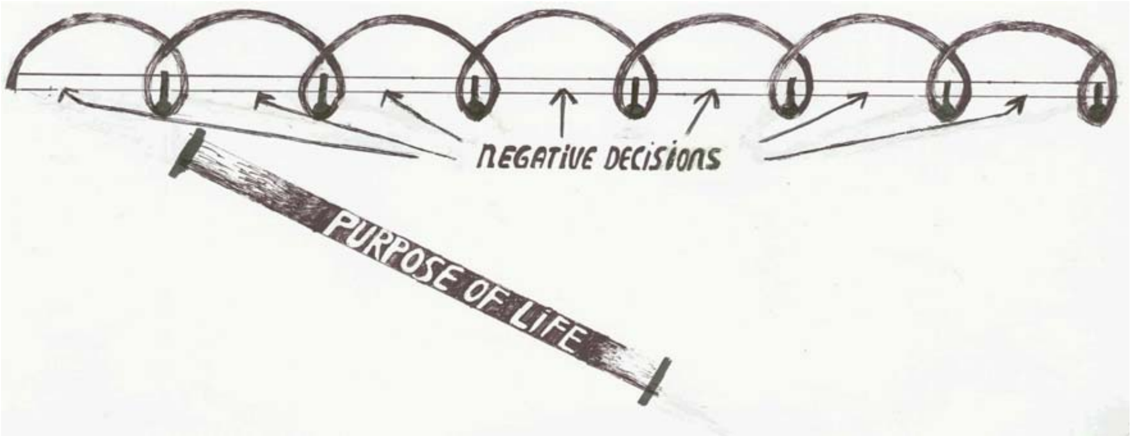

**FIGURE 3**. Low quality of life; poor health and poor functional ability in relation to social life, work life, and sexuality are derived from locking up your existence with negative decisions. The condition is rigid and undynamic and can be illustrated by a stretched spring that lost its ability to dance and vibrate freely.

When considering children, it is a little more complicated because children usually still have parents that are not completely competent holders. When parents solve their existential problems in their own lives, which often prevent them from giving the child the necessary holding, the children will normally get back their quality of life, health, and functional ability. Often it is much more efficient to help parents be better parents, than to work directly with the children. Often, one hour of competent holding of the child by the doctor demonstrates what is needed sufficiently to give the parents a better idea of competent parentship, and this alone can solve many of the problems for the child.

## CONCLUSIONS

If the physician adapts the necessary skills in holding — acknowledgement, awareness, respect, care, and acceptance — meeting the trusting patient is often enough to make the patient spontaneously go into the holistic process of healing. This process is characterized by a certain feeling of "existential movement" also known from a crisis (a feeling much like if the ground — the basis of the whole patients life — is moving). The existential movement of healing is just the opposite of the movement of crisis and adaptation for survival. In principle any problem caused by disturbances of the cells and tissues by "blockages" can be healed.

From the most abstract, holistic perspective, every problem, illness, or suffering related to the wholeness of the person basically needs the same holistic treatment: The five-dimensional holding and intention of the physician, that brings the patient into the state of existential healing. This condition is often very intense because it has the same intensity as the original trauma that forced the patient into modifying himself using the immense power of decisions.

The state of healing is not painful if the holding supplies the patient with the necessary resources. Even with the most severe traumatic life events, the confrontation of the most severe emotional pain will often be over in a few seconds or minutes if all the patient's resources are fully engaged in the process of healing. Sometimes the holding process needs more persons than just the physician or therapist, and sometimes an expanded amount of time (days) is needed for the patient to initiate, go through, and conclude the process of returning to his more natural state of being.

If the intention by the patient or the physician is unclear or a lack of correct holding, the process of treatment can drag on for a long time or may even prevent the patient from recovering or getting healed. If the physician does not succeed to get the patient through the holistic process of healing, this is usually because the physician or patient needs a more positive philosophy of life, a more safe environment, more personal resources, clearer intentions, more trust, or a more substantial and competent holding from the physician, nurse, and other employees. If the problem is with the physician, this can often be rectified through the use of existentialistic, oriented therapy.

## ACKNOWLEDGMENTS

This study was supported by grants from The 1991 Pharmacy Foundation, as well as by supplementary grants from Goodwill-fonden, the JL-Foundation, E. Danielsen and Wife's Foundation, Emmerick Meyer's Trust, the Frimodt-Heineken Foundation, the Hede Nielsen Family Foundation, Petrus Andersens Fond, Wholesaler C.P. Frederiksens Study Trust, Else & Mogens Wedell-Wedellsborg's Foundation and IMK Almene Fond. We gratefully acknowledge the critical scrutiny and expert linguistic assistance of Ib Ravn, Ph.D. The research was approved by the Copenhagen Scientific Ethical Committee under number (KF)V.100.2123/91.

## BIOSKETCHES

**Søren Ventegodt, MD,** is the Director of the Quality of Life Research Center in Copenhagen, Denmark. He is also responsible for a Research Clinic for Holistic Medicine in Copenhagen and is a popular speaker throughout Scandinavia. He has published numerous scientific or popular articles and a number of books on holistic medicine, quality of life, and quality of working life. His most important scientific contributions are the comprehensive SEQOL questionnaire, the very short QoL5 questionnaire, the integrated QOL theory, the holistic process theory, the life mission theory, and the Danish Quality of Life Research Survey, 1991–94 in cooperation with the University Hospital of Copenhagen and the late pediatric professor Bengt Zachau-Christiansen. E-mail: ventegodt@livskvalitet.org. Website: www.livskvalitet.org/

**Niels Jørgen Andersen, MSc,** Professor, Department of Innovation and Economic Organization, Norwegian School of Management. This department conducts research and provides teaching in central topics related to innovation, business development, management of global companies, business history, and economic organization. Research activities within the Department are related to four core subjects within the discipline: business history, cooperative organizations, business development and entrepreneurship, and finally studies of industries with a special focus on the electricity industry. He is also the dynamic chairman of the nonprofit organization Stiftelsen Holistisk Medisin Scandinavia, which aims to support the scientific development, research, and documentation of complementary and holistic medicine in Scandinavia. E-mail: niels.j.andersen@bi.no. Website: www.bi.no/users/fgl93013/

**Joav Merrick, MD, DMSc,** is Professor of Child Health and Human Development affiliated with the Zusman Child Development Center and Division of Community Health at the Ben Gurion University, Beer-Sheva, Israel and presently the Medical Director of the Division for Mental Retardation, Ministry of Social Affairs, Jerusalem and the Director of the National Institute of Child Health and Human Development. He has numerous publications in the field of child and human development, rehabilitation, intellectual disability, disability, health, welfare, abuse, advocacy and prevention. Dr. Merrick received the Peter Sabroe Child Award for outstanding work on behalf of Danish Children in 1985 and the International LEGO-Prize ("The Children's Nobel Prize") for an extraordinary contribution towards improvement in child welfare and well being in 1987. E-mail: jmerrick@internet-zahav.net. Website: www.nichd-israel.com
